# Impact of malocclusion on oral health related quality of life in young people

**DOI:** 10.1186/1477-7525-11-25

**Published:** 2013-02-26

**Authors:** Yaghma Masood, Mohd Masood, Nurul Nadiah Binti Zainul, Nurhuda Binti Abdul Alim Araby, Saba Fouad Hussain, Tim Newton

**Affiliations:** 1 Centre of Studies for Oral & Maxillofacial Diagnostic and Medicine, Faculty of Dentistry, Universiti Teknologi MARA, Shah Alam, Malaysia; 2 Centre of Studies for Community Dentistry, Faculty of Dentistry, Universiti Teknologi MARA, Shah Alam, Malaysia; 3 Faculty of Dentistry, Universiti Teknologi MARA, Shah Alam, Malaysia; 4 Centre of Studies for Orthodontics, Faculty of Dentistry, Universiti Teknologi MARA, Shah Alam, Malaysia; 5 Unit of Dental Public Health and Oral Health Services Research, King’s College Dental Institute, King’s College London, Denmark Hill, London, UK

**Keywords:** Oral health related quality of life, Malocclusion, Treatment need, OHIP

## Abstract

**Background:**

The objectives for this study were to assess Oral Health Related Quality of Life (OHRQoL) in young people aged 15–25 who sought orthodontic treatment, and to measure the association between orthodontic treatment need (using the IOTN), sex, age and education level, and oral health related quality of life (OHRQoL).

**Methods:**

Survey of a consecutive series of 323 young adults aged 15 to 25 years, attending orthodontic clinics at the Faculty of Dentistry, Universiti Teknologi MARA. Participants completed the Oral Health Impact Profile-14 (OHIP-14) and had a clinical examination including the Index of Orthodontic Treatment Need- Dental Health Component (IOTN-DHC). Data analyses included descriptive statistics, One-way ANOVA and bivariate and multivariate regression models.

**Results:**

The mean overall score (± SD) for OHIP-14 in young people aged 15–25 was 22.6 ± 12.5. The psychological discomfort domain was the domain where highest impact was recorded with a mean (± SD) of 4.0 ± 1.9. The regression analyses showed a significant association of IOTN-DHC with overall OHIP-14 score (p < 0.05). Although females reported a slightly higher impact than males, this was not significant in both bivariate and multivariate analyses. Age group had a significant negative association with overall OHIP-14 score (p < 0.05). The 15–18 year old group showed the highest impact on their quality of life due to malocclusion. Participants with a university education report a significantly higher impact on OHRQoL as compared to participants with only secondary education.

**Conclusion:**

Malocclusion has a significant negative impact on OHRQoL and its domains. This is greatest for the psychological discomfort domain. Younger people and those with a university education report higher levels of impact. There was no reported difference in impact between male and females.

## Background

The concept of Oral Health-Related Quality of Life (OHRQoL) corresponds to the impact of oral health or disease on an individual’s daily functioning, well-being or overall quality of life [1]. Conditions affecting oral health, including malocclusion, are highly prevalent, and have consequences not only for physical and economical well being, but can also impair quality of life by affecting function, appearance, interpersonal relationships, socializing, self-esteem and psychological well-being [2]. Research on the physical, social, and psychological impact of malocclusion on OHRQoL sheds light on the effects of malocclusion on people’s lives and provides a greater understanding of the demand for orthodontic treatment beyond the measurement of clinical parameters. In addition, since social and psychological effects are often the key motives for seeking orthodontic treatment, OHRQoL can be considered the best measurement for orthodontic treatment need and outcome [2]. Such research may be of great value to researchers, health planners, and oral health care providers [3].

Malocclusion differs from the majority of medical and dental conditions in that it is ‘a set of dental deviations’ rather than a disease, and orthodontic treatment does not cure a condition but rather corrects variations from an arbitrary norm [4]. This has led to debate about defining the point at which the extent of variation means that orthodontic treatment is desirable [5]. Further it has been suggested that the majority of oral health measures developed in dentistry are not applicable to orthodontic patients because most malocclusions are asymptomatic and related to esthetic challenges, as opposed to loss of function [4,6]. Additionally, a malocclusion can be perceived differently by the affected person, and a person’s degree of awareness of their malocclusion might not be related to its severity [3]. Therefore, when evaluating the impact of a malocclusion, it is important to consider the different domains that can be affected and their relationships to the severity of malocclusion. Some people with a severe malocclusion are satisfied with or indifferent to their dental esthetics, whereas others are concerned about minor irregularities [3].

Need assessment for orthodontic treatment in dental clinics is traditionally normatively assessed using measures such as the Index of Orthodontic Treatment Need (IOTN [3,7]. However there is evidence that many adolescents with normative orthodontic treatment need measured by IOTN experience no impacts on their OHRQoL [8,9]. Therefore, the use of IOTN alone to establish orthodontic treatment need may be potentially problematic since some patients who have no psychosocial need for treatment would be perceived as requiring treatment [10,11]. The use of OHRQoL measures in addition to professional indices offers a potentially useful combination [7,12]. OHIP-14 can be used as a potential proxy measure to replace subjective clinical opinions for determining treatment need in youth [13].

Previous research exploring the relationship between malocclusions and OHRQoL, as well as the impact of orthodontic treatment on OHRQOL has been equivocal. Some authors found a strong relationship between malocclusion or orthodontic treatment need and OHRQoL [12,14,15], but others reported no clear relationship [4,16,17]. Therefore, this study was conducted to assess OHRQoL in young people aged 15 to 25 years who sought orthodontic treatment in the Department of Orthodontics at Universiti Teknologi MARA Malaysia and to measure the association between orthodontic treatment need (using the IOTN), sex, age and education level, and OHRQoL.

## Methods

### Study design

Participants in the study were children and young adults aged 15 to 25 years, attending orthodontic clinics at the Faculty of Dentistry, Universiti Teknologi MARA, in Malaysia. The orthodontic clinic at Faculty of Dentistry UiTM is the biggest orthodontic clinic in Shah Alam city and nearby areas. It is a government clinic and all the patients who are judged to have a clinical need or who request for orthodontic treatment are referred to the Orthodontic clinic at Faculty of Dentistry UiTM.

All children and young adults aged 15 to 25 years, who either self-referred or were referred by their general dental practitioners to the orthodontic clinics at the Faculty of Dentistry, Universiti Teknologi MARA, in Malaysia. Most participants were motivated by their parents to seek orthodontic consultation. A convenience consecutive sampling approach was used. The participants were recruited at their first visit for orthodontic screening before starting any orthodontic treatment. The participants or where appropriate their parents were fully informed of the nature of the study and signed a consent form agreeing to participate in the study. To be eligible, the participant had to be in good general health. Participants who required a surgical intervention or who had chronic medical conditions, had received previous orthodontic treatment, had severe dentofacial anomalies such as cleft lip and palate, untreated dental caries, or poor periodontal health status as indicated by a community periodontal index score of 3 or more were excluded. This was to prevent possible confounding effects of these conditions on the participants’ quality of life and to achieve a homogeneous group population. The Universiti Teknologi MARA (UiTM) Research Ethics Board approved all study procedures.

### Outcome variable (OHIP-14)

OHRQoL was measured using a Malay language translated version of the 14-item Oral Health Impact Profile (OHIP-14). It has been shown that the OHIP-14 has good reliability, validity, and precision [18,19]. The Malay version of OHIP-14 has also been found to be valid and reliable and had been used in a nationally representative survey to determine population estimates for prevalence, extent, and severity of impact on OHRQoL [20]. OHIP-14 assesses the burden of oral health status on life quality across seven conceptual domains (two items per domain) by asking respondents to rate the frequency of occurrence of a particular problem as captured by the individual item. The dimensions are: functional limitation, physical pain, psychological discomfort, physical disability, psychological disability, social disability, and handicap [21]. These dimensions are based on Locker’s conceptual model of oral health [22]. Responses are rated on a 5-point Likert scale: 0 = never; 1 = hardly ever; 2 = occasionally; 3 = fairly often; 4 = very often/every day. Summary OHIP-14 scores can range from 0 to 56 and are calculated by summing the ordinal values for the 14 items. Domain scores can range from 0 to 8. Higher OHIP-14 scores indicate worse, and lower scores indicate better, oral health-related quality of life. All participants completed the OHIP-14 before any orthodontic treatment.

### IOTN-DHC

After participants were interviewed using OHIP-14, clinical examinations were conducted to assess normative orthodontic treatment need using the Dental Health Component (DHC) of the Index of Orthodontic Treatment Need (IOTN). This index has gained international acceptance because it is valid, reliable and easy to use [23]. For the Dental Health Component (DHC) of IOTN, 10 traits of malocclusion are assessed: overjet, reverse overjet, overbite, open bite, cross bite, crowding, impeded eruption, defects of cleft lip and palate as well as any craniofacial anomaly, Class II and Class III buccal occlusions, and hypodontia. Only the highest scoring trait is used to assess treatment need [24]. The treatment needs of the patients were categorized as Grade 1 (no treatment need), Grade 2 (little treatment need), Grade 3 (borderline need), and Grade 4 and 5 (high treatment need). Age, sex, and educational background were recorded because of their potential associations with both the outcome and explanatory variables.

### Examiner reliability

IOTN-DHC ratings were recorded independently by two trained and calibrated examiners. To assess intra- and inter-examiner reliability, 20 young people who were not part of the present study were randomly selected and re-examined at a 2 to 4 week interval after their first examinations. Intra-examiner reliability for the IOTN-DHC examiners was almost perfect with kappa = 0.91 and 0.96. Excellent agreement was found for the inter-examiner reliability with Kappa = 0.85.

### Statistical analysis

The data were analyzed by using R-Project software (version 2.13.2) [25]. Additive scale and subscale scores for the OHIP-14 were calculated by summing the item response codes. Data analyses included descriptive statistics and One-way ANOVA with Tukey Post Hoc test to assess differences in OHRQoL across groups defined by IOTN-DHC. Bivariate and multivariate regression models were used to measure the association between OHRQoL and IOTN-DHC, sex, age and education level. In linear regression analysis, OHIP-14 scores were used as continuous outcome variable, IOTN-DHC was recoded as a series of dummy variables. Age was recoded into three categories, and dummy variables calculated for use in the regression analyses. Bivariate linear regressions were performed individually in separate models with each explanatory variable; model 1 included IOTN-DHC (No treatment required = 0, little treatment required = 1, borderline treatment required = 2 and high treatment required = 3), model 2 included gender (Male = 0 and Female = 1), model 3 included age groups (15–18 years = 0, 19–21 years = 1, 22–25 years = 2) and model 4 included educational level (secondary education = 0 and university education = 1). Finally, all the explanatory variables were combined into a multivariate linear regression model.

## Results

A total of three hundred and twenty five young adults participated in this study, two records were excluded from analysis due to missing values in their OHIP-14 questionnaires. The mean (± SD) age was 22.05 (± 3.20) years which included 63 participants (19.5%) aged 15–18 years, 89 (27.5%) aged 19–21 years and 171 (53.0%) in the 22–25 age group. Most of the participants (273, 85.4%) had education to university level and 49 (15.1%) were educated only to secondary level. Of the 323 participants, 132 (40.8%) were boys and 191 (59.1%) were girls. Normative treatment need according to IOTN-DHC was present in 252 (78.0%) participants, the remaining 71 participants (22%) did not have any normative treatment need for orthodontic treatment as assessed by the IOTN-DHC.

Table 1 displays the mean, standard deviation, median and the range for the total OHIP-14 score and for each domain in all 322 participants. The mean overall score (± SD) for OHIP-14 was 22.6 ± 12.5. The psychological disability (2.6 ± 1.8) domain of the OHIP-14 showed the least impact due to malocclusion in patients with perceived need for malocclusion. Whereas, the psychological discomfort domain had the highest reported impact with a mean (± SD) 4.0 ± 1.9.

**Table 1:** **Mean**, **standard deviation **(**SD**), **median and range observed in Oral Health Impact Profile**- **14 **(**OHIP-14**)

**OHIP domain**	**Mean** **±** **SD**	**Median**	**Range observed**
Functional limitation	3.6 ± 2.1	4	0-8
Physical pain	3.3 ± 1.7	4	0-7
Psychological discomfort	4.0 ± 1.9	4	0-8
Physical disability	3.2 ± 2.0	4	0-8
Psychological disability	2.6 ± 1.8	2	0-7
Social disability	2.7 ± 2.1	3	0-6
Handicap	3.2 ± 2.0	4	0-6
OHIP-14 total	22.6 ± 12.5	25	0-46

Table 2 shows the results from the Oneway ANOVA and post hoc Tukey test comparing groups defined by IOTN-DHC. Participants with high treatment need reported a significantly greater negative impact on the overall OHRQoL score and in each domain of OHIP-14. The greatest impact was seen in the psychological discomfort domain where even having little treatment need was associated with a significant difference in OHIP-14 scores in comparison to the "No treatment need" group. Whereas, the domains of functional limitation, physical pain, physical disability, psychological disability and social disability all showed a significant difference in OHIP-14 score at the level of the “borderline treatment need” group, the handicap domain only showed a significant difference in OHIP-14 scores in the “high treatment need” group.

**Table 2:** **Mean scores in overall and seven domains of Oral Health Impact Profile-14 **(**OHIP-14**) **OHIP-14 among different types of Index of Orthodontic Treatment Need- Dental Health Component **(**IOTN-DHC**) **groups**

**IOTN**-**DHC**^**1**^	**N (%)**	**Functional limitation**	**Physical pain**	**Psychological discomfort**	**Physical disability**	**Psychological disability**	**Social disability**	**Handicap**	**OHIP-14 total**
No treatment need (Reference Group)	71 (22.0%)	2.2 ± 1.8	2.1 ± 1.6	2.6 ± 1.8	2.0 ± 1.6	2.0 ± 1.6	1.8 ± 1.6	2.4 ± 1.7	15.2 ± 10.9
Little treatment need	87 (26.9%)	3.2 ± 1.9	3.0 ± 1.6	3.8 ± 1.8***	3.0 ± 1.9	2.0 ± 1.6	2.2 ± 2.0	2.9 ± 1.8	20.2 ± 11.1*
Borderline treatment need	80 (24.8%)	3.9 ± 2.0**	3.4 ± 1.3**	4.5 ± 1.6***	3.4 ± 1.9*	2.9 ± 1.7*	2.9 ± 1.9*	3.2 ± 1.9	24.1 ± 11.2**
High Treatment need	85 (26.3%)	4.8 ± 2.0***	4.2 ± 1.7***	4.9 ± 1.7***	4.0 ± 2.0***	3.6 ± 1.9***	3.8 ± 2.2***	4.1 ± 2.0**	29.5 ± 12.5***

* P < 0.05, ** P < 0.01 and ***P < 0.001.

^1^ Group comparisons were performed by One Way Analysis of Variance (ANOVA) and Tukey Post Hoc test.

Table 3 shows the results of the bivariate and multivariate analyses to establish the association between OHIP-14 and IOTN-DHC, gender, age group and education level. There is a significant association of normative orthodontic treatment need with overall OHIP-14 score (p < 0.05). Although females have slightly higher impact scores than males, this is not significant in either analysis. Age group is negatively associated with overall OHIP-14 score (p < 0.05). The 15–18 year old group showed the highest impact on their quality of life due to malocclusion. This age group association becomes stronger in the multivariate model. The association between education level and IOTN was not significant in the bivariate model, but became significant in the multivariate model, participants with a university education report a significantly higher impact on OHRQoL as compared to participants with only secondary education. The final multivariate model explained 22% variation in the total score of OHIP-14 (R^2^ =0.22).

**Table 3:** **Bivariate and multivariate linear regression models showing association of Oral Health Impact Profile-14** (**OHIP-14**) **with Index of Orthodontic Treatment Need-Dental Health Component** (**IOTN-DHC**) **and other covariates**

	**Bivariate analysis**			**Multivariate analysis**	
	**Estimates**	**SE**	**R**^**2**^	**Estimates**	**SE**
**IOTN**-**DHC**					
Intercept	15.239	1.37***	0.16		
No treatment need	Reference Group			Reference Group	
Little treatment need	4.97	1.84**		4.77	1.79**
Borderline treatment need	8.88	1.88***		9.03	1.83***
High Treatment need	14.27	1.85***		14.41	1.80***
**Sex**					
Intercept	21.9	0.90***	0.003		
Male	Reference Group			Reference Group	
Female	1.47	1.41		1.36	1.28
**Age**					
Intercept	27.06	1.55***	0.03		
15 - 18 years	Reference Group			Reference Group	
19 - 21 years	−4.01	2.03*		−6.12	2.22**
22 - 25 years	−6.40	1.81***		−9.46	2.12***
**Education Level**					
Intercept	23.44	1.7***	0.009		
Secondary education	Reference Group			Reference Group	
University education	−1.05	1.9		4.45	2.24*
				R^2^ = 0.22	

* P < 0.05, ** P < 0.01 and ***P < 0.001.

## Discussion

This study evaluated the impact of orthodontic treatment need on overall OHRQoL and its various domains in 15–25 year olds seeking orthodontic treatment at UiTM Dental clinics. Nearly 1.5 times as many girls as boys sought orthodontic treatment at the university clinic during the recruitment period for this study, this agrees with previous studies which show higher utilization of orthodontic treatment services by females [3,15,26,27]. This may suggest that women are responding to social expectations of the importance of esthetics rather than an objectively greater orthodontic treatment need in comparison to their male counterparts [28]. Alternatively parents may be more prone to seek orthodontic treatment for their female children than their male. It was expected that the impact of malocclusion on quality of life would be significantly greater in girls as compared to boys, since boys may be less self-conscious about their appearance [28]. Young women were more likely to have had a higher dental impact than male youths but the difference was not significant. Similar findings were reported by Oliveira and Sheiham (29) in adolescents [29] and other age groups by Birkeland et al., Hunt et al., 57 and Bernabe ´et al. [8,30,31]. However, Peres (2008) et al. found females adolescents had greater dissatisfaction with their dental appearance [32]. It is possible that the lack of a difference in impact between males and females in this and other studies is due to selection: the reluctance of males to attend clinics resulting in those who feel little or no impact not attending.

Bivariate and multivariate regression analysis showed that those participants with more severe malocclusions reported a greater impact on quality of life after controlling for the effects of covariates (sex, age, and education level). Young people with little, borderline and high orthodontic treatment need had 5, 9 and 15 points higher scores on the OHIP-14 scale, respectively, when compared to the "no treatment need" group. This relationship between malocclusions and OHRQOL mirrors that found by Heravi et.al., Bernabe et. al. and Foster-Page et. al. [8,14,28], but not by Taylor et al. or Oliveria et al. (2008)[16,33]. There are several possible reasons for such differences. First, the use of different measures to measure quality of life. Secondly it is possible that different age groups have a different perception towards esthetics and quality of life, and studies have varied widely in the age groups studied. In addition, different cultures, traditions, and social norms across countries may influence the perception of esthetics different in each society. Finally, a high frequency or severity of malocclusions in some races and ethnic groups can make malocclusion perceived as normal for the given group and vice versa [28].

A negative association was observed between age and impact on quality of life due to malocclusion, the impact of malocclusion decreases as age increases. This may be the result of ‘response shift’ with age – the longer an individual lives with a malocclusion, the greater the likelihood that they will adjust to the limitations it places upon their activities thereby reducing impact. In order to explore this, future studies should look at treatment need changes with age. Education level had a positive association in the multivariate regression analysis which may be due to increased self awareness and self esteem with increasing education.

Exploring each OHIP-14 domain, IOTN scores were most closely correlated with impact on the psychological discomfort and functional limitation domains of OHRQoL. Similar results have been reported in children aged 11–14 years [4,14] and in young adults [3,29]. This is logical when we consider that the most common reason for seeking orthodontic treatment is to correct dental esthetics and improve self-esteem [3]. Thus, orthodontists should be aware that young patients might expect orthodontic treatment to provide not only improved oral functioning and health but enhancement of esthetics, self-esteem and social life [34]. When these expectations are not met this may lead to dissatisfaction with treatment outcome. The use of the OHRQoL measure as a part of the diagnostic procedure can provide information on priorities for treatment in order to maximize patient satisfaction [3,12,35]. In this study, significant impact on the handicap domain was only present when patients had high treatment need. Some authors have suggested that malocclusion might become handicapping not because of the functional disability, but because it can adversely affect social relationships and self-perceptions [36].

The findings of this study must be tempered by a consideration of its limitations. The participants in this study were 15–25 year-old patients seeking orthodontic treatment at a university clinic in a large urban city in Malaysia. Therefore the results from this study cannot be extrapolated to the entire youth population who may have differing levels of malocclusion and orthodontic treatment needs and, therefore, different impacts on their daily activities [12]. Although many studies have assessed the impact of oral conditions on children’s quality of life using convenience samples in hospitals or universities [1,4,14], future studies should be based on a representative sample with or without normative need. Furthermore participants may have exaggerated the impact in order to increase their chances to obtain treatment for their malocclusion.

OHIP-14 and IOTN-DHC were used in this study, both of these instruments are valid and reliable but have some limitations [3]. IOTN may be a relatively insensitive instrument to measure minor occlusal traits and irregularities which mostly affect patient appearance and about which a patient is deeply concerned [8,10]. The OHIP-14 was developed for adults, but has been successfully applied to adolescents by many authors [3,27,29,37] because adolescents of 12 years of age and above are capable of abstract thinking, reasoning about the timing of past events, and relating them with good or bad experiences [3]. Another limitation with OHIP-14 is, it does not elicit the specific cause(s) of the impacts recorded, which can be related to a variety of oral health conditions and not necessarily the subject’s malocclusion. However the participants in this study were selected to be free of untreated caries, periodontal disease and any other oral health problem suggesting the results form OHIP-14 were not confounded with other oral health condition.

## Conclusion

Malocclusion has a negative impact on oral health related quality of life and its domains, this is highest for the psychological discomfort domain. Reported impact is greatest in younger people and those with a university education, whereas this study did not find any significant difference in the impact on oral health related quality of life in male and females.

## Competing interests

The authors declare that they have no competing interests.

## Authors’ contributions

All the authors approved the final draft of the manuscript. YM contributed to data analysis and manuscript writing; NBZ and NBA contributed to participants recruitment and data collection. SFH helped in malocclusion data collection. TN and MM contributed to the conceptual framework of the project. 
